# A Review of Novel Die Attach Materials for High-Temperature WBG Power Electronic Applications

**DOI:** 10.3390/ma18163841

**Published:** 2025-08-15

**Authors:** Na Wu, Yuxiang Li

**Affiliations:** School of Mechanical and Automotive Engineering, Qingdao University of Technology, Qingdao 266520, China; yuxiang_li1997@163.com

**Keywords:** WBG, high-temperature, TLP, TLPS, nanoparticle sintering

## Abstract

Third-generation wide-bandgap (WBG) semiconductor power electronics exhibit excellent workability, but high-temperature packaging technology limits their applications. TLP, TLPS, and nanoparticle sintering have the potential to achieve a high-temperature-resistant joint at a lower bonding temperature. However, a long bonding time, voids in the joint, powder oxidation, and organic solvent residues impede their application. A novel interlayer and other approaches have been proposed, such as preformed Sn-coated Cu foam (CF@Sn), a Cu-Sn nanocomposite interlayer, self-reducible Cu nanoparticle paste, bimodal-sized Cu nanoparticle pastes, organic-free nanoparticle films, and high-thermal-conductivity and low-CTE composite paste. Their preparation, bonding processes, and joint properties are compared in this paper.

## 1. Introduction

Third-generation wide-bandgap (WBG) semiconductor materials such as SiC and GaN exhibit characteristics such as a high-breakdown electric fields, high saturation electron velocity, high thermal conductivity, and high electron density [1,2]. Their power electronics maintain excellent operational capability even at a temperature of 500 °C, meaning they have significant application potential in fields such as high-power microwave weapons, 5G/6G communication, new-energy vehicles, and ultra-high-voltage power transmission and transformation [3,4,5,6]. However, the maximum service temperature of WBG power electronics is limited by its packaging technology. Therefore, it is important to achieve high-temperature-resistant and reliable connections between WBG power chips and substrates [7]. This is a critical issue that must be urgently addressed to sufficiently take advantage of their potential uses.

Transient liquid-phase (TLP) bonding has been identified as a promising solution to surmount this challenge. A high-temperature-resistant joint that is fully composed of intermetallic compounds (IMCs) can be obtained by solid–liquid interdiffusion/reaction between high-melting-point metals and low-melting-point metals [8]. The re-melting temperature of the joint is higher than the bonding temperature, leading to a higher service temperature. However, TLP bonding suffers from several inherent limitations, including long reaction time, coarse grain formation, and Kirkendall voids. In earlier research, ultrasonic vibration [9,10,11,12,13] and electric current assistance [14,15,16] have been used to reduce TLP bonding time. However, high-power ultrasonic vibration and high-density electric current may cause damage to the WBG power electronics [17,18].

Against this background, TLP sintering with mixed powders has received considerable attention [19]. The structures of high-melting-point metals and low-melting-point metals transform from laminated multilayers to mixed powders. The solid–liquid reaction area is enlarged, and the diffusion distance is decreased, which can accelerate the diffusion reaction and the isothermal solidification process. However, certain problems still remain. Most TLPS powders exhibit high susceptibility to oxidation and poor mixing homogeneity. Low-melting-point metal powders fail to sufficiently fill porosities between high-melting-point particles, resulting in a long reaction time and voids in the brazed region. Sintering pressure can help to reduce the voids, but excessive pressure leads to solder expulsion and chip damage. Moreover, the production of fine powders is complex, and the cost of production is high. Thus, a Cu@Sn core/shell powder was prepared for TLPS [20,21,22,23], which can protect small particles from oxidation and improve mixture uniformity.

In early reviews about high-temperature-resistant packaging technology for wide-bandgap power electronics, transient liquid-phase bonding (TLP), transient liquid-phase sintering (TLPS), and low-temperature silver nanoparticle sintering have been proposed [24]. The initial interlayer materials for TLP had a laminated sheet structure, while Cu particles and Cu-Sn core-shell particles were employed in TLPS. In the low-temperature silver sintering process, Ag nanoparticles and Ag-Cu nanoparticles were used. The problem of a long bonding time was addressed by using a thinner laminated thickness, electric current assist, and ultrasonic assist [8]. Reviews [25,26,27,28] were mainly focused on the microstructure evolution, interfacial reaction, bonding mechanism, void formation, reliability, and properties of full IMC solder joints [29].

Nowadays, the structure of the interlayer is no longer limited to laminated multilayers or mixed powders. In this paper, novel interlayer materials and structures in the past five years are summarized. The preparation process, bonding technology, and joint property are reviewed as well.

Various novel approaches have been proposed to increase the contact area between low-melting and high-melting components, such as preformed Sn-coated Cu foam (CF@Sn) [30], preformed Cu foam/SAC305 composite solder [31], Cu-Sn nanocomposite interlayers [32], and Sn-Cu-coated multi-walled carbon nanotubes (MWCNTs) [33].

Nano-Ag sintering technology is also a high-temperature-resistant packaging technique that utilizes nano-Ag particles to achieve bonding at relatively low temperatures. Due to the size effect, nano-Ag particles exhibit high surface activity and surface energy even at room temperature, enabling sintering at temperatures much lower than those required for bulk Ag.

Ag exhibits excellent electrical conductivity and oxidation resistance. However, it is prone to causing electromigration and may even short-circuit under high-temperature or humid conditions. This limits the application of nano-Ag sintering in high-power semiconductor packaging [34]. Cu possesses outstanding electrical and thermal conductivity. However, its susceptibility to oxidation necessitates sintering under a vacuum or in a reducing atmosphere. Therefore, a core–shell-structured particle with a Cu core and a Ag shell was proposed [35]. Nevertheless, it is difficult to suppress copper oxidation completely due to the Ag shell dewetting phenomena at the Cu core interface. Furthermore, to prevent particle agglomeration, nanoparticles must be dispersed in organic solvents, which tend to generate pores in the joint due to incomplete removal during sintering. Therefore, nanoscale Ag and Cu thin films were deposited on both the substrate surface and chip backside via magnetron sputtering, forming organic-free nanoparticle films for low-temperature sintering [36,37].

## 2. TLP Bonding

During TLP bonding, the interlayer (composed of low-melting-point materials) undergoes melting, and the interlayer element (or a component of an alloy interlayer) diffuses into the substrate (consisting of high-melting-point materials). This process leads to isothermal solidification accompanied by the formation of intermetallic compounds (IMCs) [8]. The remelting temperatures of the IMCs exceed the bonding temperature, rendering the fully formed IMC joints stable in high-temperature environments. Furthermore, the mechanical properties of the full IMC joints are superior to those of Sn-rich joints [38]. TLP bonding has attracted significant attention due to its cost-effectiveness and straightforward process.

Rautiainen et al. [39] electroplated micron-scale Au and Sn layers onto the surfaces of Si wafers. They achieved a fully intermetallic compound (IMC) joint through TLP bonding at 320 °C under a pressure of 2.4 MPa. Nevertheless, due to the slow diffusion/reaction rate between the Au and Sn layers, the bonding process took as long as 60 min. Rodriguez et al. [17] deposited Au layers and Au80Sn20 (wt.%) alloy layers on the surfaces of chips and substrates via vapor deposition. After heating at 340 °C for 5 min, fully IMC joints were obtained. Compared with single-component Au and Sn layers, the Au-Sn alloy layers proved able to significantly shorten the reaction time. Tollefsen et al. [40] bonded Au-plated (5 μm) SiC chips and Si_3_N_4_ substrates using 7.5 μm Au80-Sn20 preform solder sheets via TLP bonding. Achieving fully IMC joints required at least 300 °C and 6 min.

### 2.1. Addition of Ni

**Figure 1:**
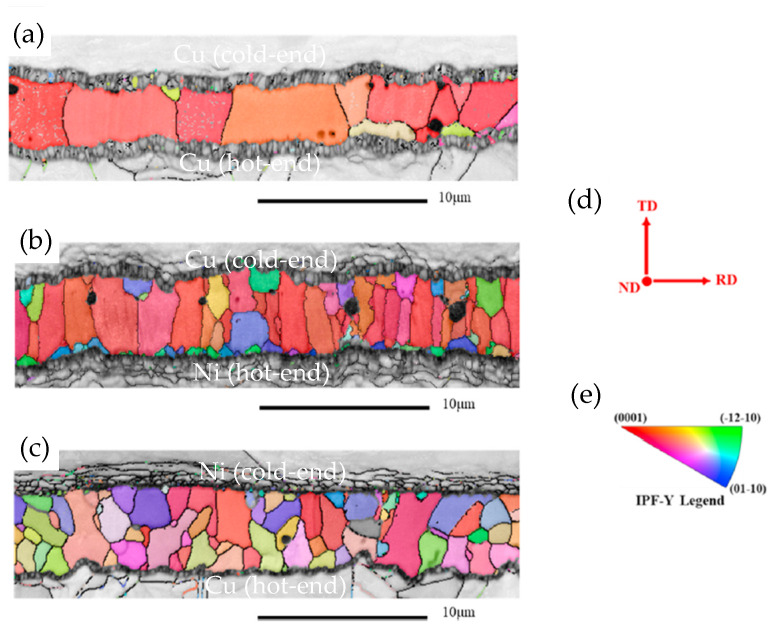
EBSD orientation maps of Cu-Sn IMCs in (**a**) Cu/SAC305/Cu, (**b**) Cu/SAC305/Ni, (**c**) Ni/SAC305/Cu TLP bond. (**d**) The direction of transverse direction TD, normal direction ND and rolling direction RD in EBSD analysis and (**e**) the inverse pole figure of the TD direction. Reprinted with permission from Ref. [42]. Copyright 2023 Elsevier.

A notable shortcoming of TLP is the extremely long time required to complete the process. This not only reduces production efficiency but also raises production costs. Additionally, the prolonged bonding time leads to preferred grain orientation and coarse grains [41], consequently resulting in a degradation of joint reliability. During the progression of the TLP reaction, Cu reacts with Sn to form Cu_6_Sn_5_. Subsequently, Cu_6_Sn_5_ undergoes further reaction with Cu, resulting in the generation of Cu_3_Sn. The Cu_3_Sn layer at the Cu_6_Sn_5_/Cu interface is also an issue for the Cu/Sn/Cu TLP joint. Kirkendall voids tend to form concurrently with the growth of Cu_3_Sn layer, increasing the brittle fracture susceptibility of the joints. As the soldering temperature rises, more voids are formed within the joints. The grain size of the intermetallic compounds (IMCs) is refined as Ni dissolves from the substrate (Figure 1) [42]. The addition of Ni at the cold end contributes to grain refinement and orientation diversification, which enhances the mechanical properties of the Cu/SAC305/Cu TLP joint.

Abdul Razak et al. [43] carried out an investigation into the growth kinetics and void formation behavior during the growth process of IMCs through in situ observations using synchrotron X-ray imaging. Different Ni concentrations in the Cu-Ni substrates can regulate the location of porosity and lead to different growth rates of (Cu,Ni)_6_Sn_5_ and Cu_6_Sn_5_ IMCs. Cu-Ni substrates with different concentrations can be utilized to minimize porosity in the joints, which can be employed to develop reliable electronic interconnections for the power device industry.

### 2.2. Addition of SiC NWs

**Figure 2:**
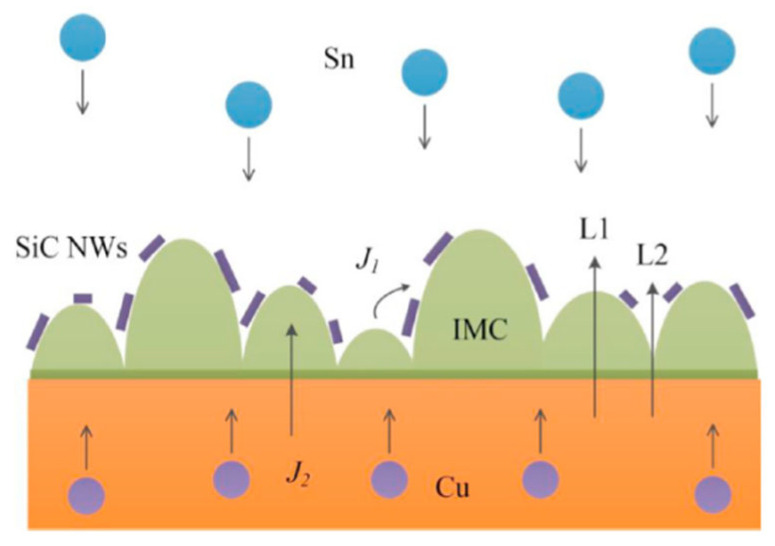
Schematic diagram of IMC growth path through the interface. Reprinted with permission from Ref. [44]. Copyright 2022 Elsevier.

Li Mulan et al. [44] incorporated SiC nanowires (NWs) into pure Sn solder to enhance the performance of the composite solder. The addition of SiC NWs accumulated on the surface of IMC, which impeded atomic inter-diffusion and reaction, as depicted in Figure 2. Consequently, the growth of Cu_6_Sn_5_ and Cu_3_Sn IMCs at both the hot and cold end interfaces was inhibited. Moreover, the number of voids among the IMCs in the solder joints with added SiC NWs was smaller than that in pure Sn solder joints. Thus, the addition of SiC NWs improved the reliability of Sn-based solder joints.

### 2.3. Pre-Soldering Annealing of Ni Pads

As mentioned in previous studies, voids between IMCs and solder are inevitable due to the Kirkendall effect. Njuki et al. [45] found that when high-purity nickel was employed as pads, no voids were detected in the solder joint. Conversely, electroplated nickel pads led to the generation of voids. During the reflow process, the outgassing of hydrogen and organic impurities triggered the nucleation of micro-bubbles. Subsequently, Kirkendall vacancies fed these bubbles and enlarged the voids. Pre-annealing electroplated nickel pads at 450 °C for 24 h effectively eliminated these impurities and completely suppressed void formation, suggesting that voids can be circumvented through appropriate process control.

## 3. Transient Liquid-Phase Sintering

Transient liquid-phase sintering (TLPS) technology is a modified TLP bonding process. It operates on the principle that when two appropriate metal powders are combined, they react to form intermetallic compounds (IMCs) with a high melting point [46,47]. By increasing the effective solid–liquid reaction area and reducing the diffusion distance, this technology can expedite the diffusion reaction and isothermal solidification processes. Consequently, TLPS offers an attractive strategy for fabricating die attach materials that can achieve “low-temperature bonding and high-temperature serviceability”, holding great promise for high-temperature electronic applications.

Jeong et al. [48] synthesized the 30Ni-70Sn (wt.%) TLPS paste. This was achieved by mixing 99.9% pure Ni powder (particle size: 1–3 μm) and Sn powder (particle size: 5–7 μm) in ethyl alcohol under vacuum conditions. The electroless nickel/immersion gold (ENIG)-finished Cu dummy chip and the ENIG-finished direct bonded copper (DBC) substrate were joined via Ni-Sn TLPS bonding. After holding at 300 °C for 15 min, the joint was composed of residual Ni particles and surrounding Ni_3_Sn_4_ intermetallic compounds (IMCs). The joint exhibited a maximum shear strength of 47.9 MPa.

Moreover, for applications in power electronics, the microstructural and mechanical stabilities of the TLPS joints under high-temperature and extremely high-temperature conditions were investigated [49,50]. During the aging process at 200 °C, Ni_3_Sn_4_ reacted with the residual Ni particles and transformed into Ni_3_Sn_2_. Even after 1000 h of aging, some residual Ni particles remained. Significantly, the shear strength of the TLPS joints showed no substantial change even after long-term aging.

When the aging temperature was elevated to 500 °C, all the residual Ni particles were consumed and converted into the Ni-rich Ni_3_Sn IMC. This transformation enhanced the integrity of the microstructure and increased the mechanical strength by approximately 2–3 times.

TLPS offers the advantage of shortening the bonding time by virtue of the large surface area of the powder. Nevertheless, certain drawbacks persist. Fine TLPS powders are highly prone to oxidation and exhibit poor mixing homogeneity [51]. The low-melting-point metal powders are unable to adequately fill the porosities, leading to an extended reaction time and the presence of voids in the sintered zone. Sintering pressure can assist in reducing voids; however, excessive pressure causes solder expulsion, thereby decreasing the joint strength. Cu substrates were bonded using a mixture of Cu nanoparticles and Sn nanoparticles at 300 °C for 5 min [22,52]. Nevertheless, due to numerous voids in the sintered region, the shear strength was less than 10 MPa. Additionally, the production of fine powder is complex and costly.

To address these limitations, numerous novel approaches have been proposed (Table 1). To enhance the contact area between the low-melting-temperature and high-melting-temperature components, various materials such as a Cu@Sn core–shell [22,23], Sn-coated copper foam [30], Cu foam/SAC305 composite solder preform [31], Cu-SnAgCu molded sheets [53], Cu-Sn nanocomposite interlayer [32], and Sn-Cu coated multi-walled carbon nanotube (MWCNT) [33] have been utilized. However, the fabrication of these materials still poses significant challenges.

### 3.1. High-Sn-Content Cu@Sn Core/Shell Powder

Regarding the primary issue of TLPS that small-sized powders are prone to oxidation and difficult to mix uniformly, Yoon et al. [57] put forward a type of Cu@Sn core/shell powder fabricated via immersion plating for bonding Cu substrates. The Sn shell can safeguard the Cu core from oxidation, and the core/shell powder does not necessitate elaborate homogeneous mixing. The shear strength of the joint sintered at 300 °C for 30 min reached approximately 40 MPa.

Nevertheless, the Sn shell formed outside the Cu core via immersion plating was relatively thin. This thin shell was unable to provide an adequate amount of liquid during the sintering process. As a result, the bonding pressure had to be increased to 15 MPa. However, such an increase in bonding pressure was detrimental to the brittle chips. Consequently, a Cu@Sn core/shell powder with a high Sn content was anticipated to address the problems associated with TLPS bonding, including prolonged bonding time, elevated bonding pressure, and high porosity.

Peng Xianwen et al. [22] synthesized Cu@Sn core/shell powders via electroless plating. Subsequently, the electroless plating procedure was reiterated three times to achieve a high Sn content of 60 wt.%. The TLPS bonding process of Cu with the Cu@Sn paste was carried out under a bonding pressure of 0.2 MPa in a vacuum (≤1.0 × 10^−3^ Pa). As the bonding time increased, the molten Sn was gradually consumed. When the bonding time reached 150 min at 300 °C, a complete IMC joint with some remaining Cu particles was attained, and the shear strength reached 20.67 MPa.

When compared with the Cu-Cu joints bonded with Cu-Sn mixed powder, the Cu@Sn TLPS joints exhibited fewer voids and higher shear strength. In the Cu-Sn TLPS bonding process, volume shrinkage resulting from the Cu-Sn reaction led to the formation of voids. The size and amount of the shrinkage were determined by the isolated molten Sn. When the formed Cu_6_Sn_5_ began to make contact and connect, the molten Sn in the bonding layer was separated by the brittle Cu_6_Sn_5_ skeleton. During the TLPS process, due to the difficulty of evenly mixing the Cu-Sn powders, the Cu particles were non-uniformly distributed in the molten Sn, and voids were prone to form in this region. In contrast, during the TLPS of Cu@Sn core/shell powders, the Cu particles were uniformly distributed in the molten Sn, which was helpful for reducing the formation of voids. Additionally, not all of the Cu particles in the Cu-Sn mixed powders were thoroughly wetted by the molten Sn, leading to the formation of voids in the brazed region [23].

### 3.2. Interlayer of Cu Powders Between Sn Foils

In the conventional TLPS process, numerous voids remain along the intermetallic grain boundaries, even when the bonding pressure is elevated to several MPa [58]. This is because there is an insufficient supply of liquid to fill the porosities between adjacent particles. Additionally, the Cu and Sn particles are non-uniformly distributed in the powder, and excessive pressure can squeeze the molten solder out of the assembly gap. Therefore, this has an adverse impact on the thermal reliability and mechanical properties.

**Figure 3:**
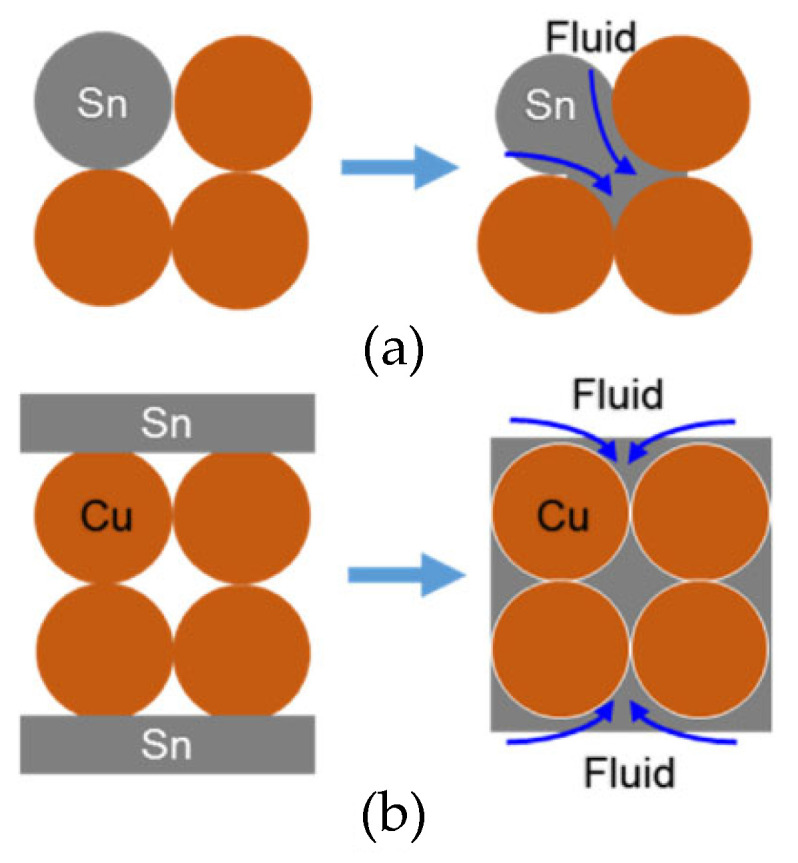
Schematic diagram of the microstructure densification in (**a**) TLP sintering and (**b**) this method. Reprinted with permission from Ref. [54]. Copyright 2018 Elsevier.

**Figure 4:**
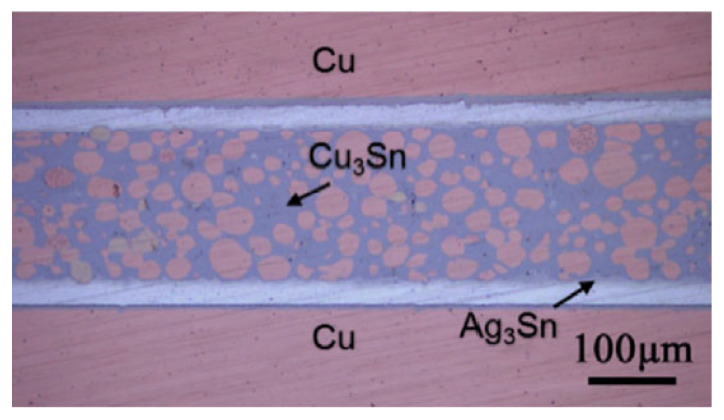
Micrograph of the joint bonded at 300 °C for 90 min with 1 MPa pressure (substrate: Cu/2 μm Ni/25 μm Ag). Reprinted with permission from Ref. [54]. Copyright 2018 Elsevier.

Shao Huakai et al. [54] fabricated a sandwiched TLPS perform by compacting a thin layer (200 μm) of Cu powders between Sn foils (20 μm). During the bonding process, the Sn foils melted and, through capillary action, flowed into the gaps between Cu particles. In comparison with TLP sintering, as depicted in Figure 3, there was an adequate liquid supply for the original gaps. It only took 4.26 × 10^−6^ s to completely fill the gaps within the porous Cu interlayer. Thus, in the early stage of the reaction, the Cu-Sn IMCs did not block the flow channels. Additionally, the filling effect was determined by the average size of the original Cu particles. The flow channels among 2 μm spherical particles and irregular particles were complex, resulting in a great amount of micro-voids in the Cu-Sn IMC layer. Spherical particles of 20 μm were more suitable for capillary action. As illustrated in Figure 4, the molten Sn effectively filled the gaps between Cu particles in the brazed region.

A maximum shear strength of 32.9 MPa was achieved at 300 °C for 45 min under a pressure of 0.3 MPa. The joint was primarily composed of Cu_6_Sn_5_, Cu_3_Sn and residual Cu particles. After aging at 350 °C for 960 h, there were no new voids, cracks, or other heat-affected defects across the interconnection layer.

### 3.3. Sn-Coated Cu Foam (CF@Sn) Preform

Core–shell structured particles, characterized by a low-melting-point Sn shell and a high-melting-point metal core, were designed to facilitate the rapid fabrication of thermally stable joints [59]. However, the micron/nanoscale dimensions of these particles intensify spontaneous oxidation, which undermines bonding strength. Moreover, the preparation of these particles remains technically challenging.

A porous-foam architecture was utilized to establish continuous flow channels and optimize the surface area for low-melting-point die attach materials. This approach aimed to maximize the solid–liquid reaction kinetics via ultrasonic activation, as reported in reference [60]. Nevertheless, incomplete infiltration of the low-melting-phase material into the porous matrix of the foam was observed. Moreover, it was discovered that the interfacial shear stress and localized thermal transients generated by ultrasonic-assisted sintering could compromise the device integrity.

**Figure 5:**
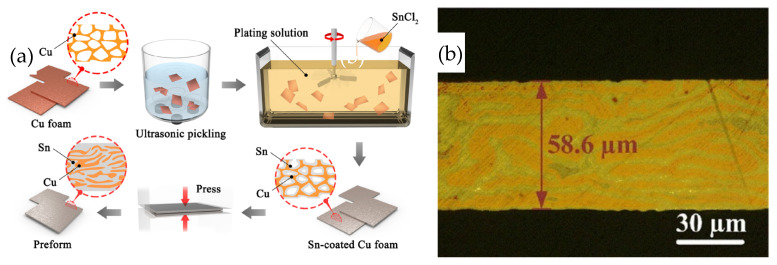
Schematic preparation process (**a**) and microstructure (**b**) of Sn-coated Cu foam preform. Reprinted with permission from Ref. [30]. Copyright 2022 Elsevier.

Liu Jiaxin et al. [30] developed a low-cost Sn-plated Cu foam (CF@Sn) preform. The structure featured Sn within the porous Cu foam, and the preparation process is depicted in Figure 5. Initially, Sn was applied through electroless plating to the strip-shaped Cu skeleton within the foam. Subsequently, the Sn-coated Cu foam was compressed into a preform at 400 MPa for 300 s.

Cu substrates coated with a 2 μm Ag layer were bonded using a CF@Sn preform at 280 °C for varying durations under a pressure of 2 MPa in Ar atmosphere. After a bonding time of 10 min, residual Sn was still present in the joint. As the bonding time was extended to 20 min, the Sn was completely consumed and converted into Cu_6_Sn_5_. Subsequently, Cu_6_Sn_5_ reacted rapidly with Cu, leading to the formation of Cu_3_Sn and the emergence of gaps and voids within the Cu skeleton. When the bonding time was further increased to 40 min, Cu_6_Sn_5_ was completely transformed into Cu_3_Sn, and no voids were observed in the joint, suggesting that a longer bonding time can promote atomic diffusion and suppress void formation. The shear strength reached a maximum value of 28.1 MPa. After aging at 300 °C for 100 h, the shear strength decreased to 25.3 MPa, which was caused by the microstructure coarsening and formation of voids in the joint.

### 3.4. Cu Foam/SAC305 Composite Solder Preform

The IMCs initially formed at the interface between the solder and the substrate. Consequently, as the bonding process progressed, elemental diffusion was required to traverse the IMC layer, resulting in a significant reduction of the diffusion rate [61].

Heo et al. [31] focused their attention on the porous structure of Cu foam. This structure featured a large surface area, which would enable more rapid reaction between Cu and Sn atoms in comparison with the conventional sandwich-type structure. Additionally, owing to the high electrical and thermal conductivities of Cu, the skeleton can serve as an electrical/thermal pathway and effectively suppress crack propagation.

**Figure 6:**
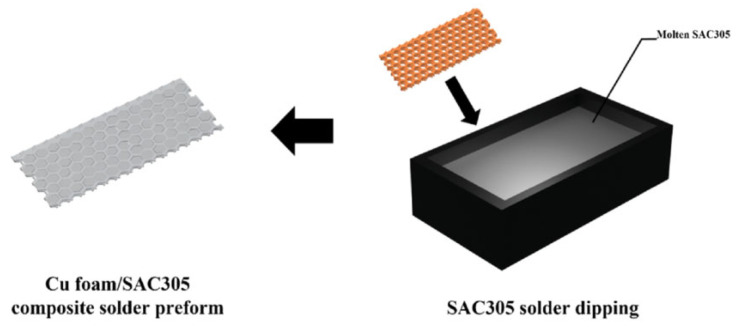
Schematics of the fabrication process of the Cu foam/SAC305 composite solder preform. Reprinted with permission from Ref. [31]. Copyright 2023 Elsevier.

To further increase the surface area of the Cu foam and its reaction rate with Sn, the Cu foam was etched in an FeCl_3_ solution at 25 °C and then immersed in a molten Sn-3.0Ag-0.5Cu (SAC305) solder bath for 1–2 s to fabricate the composite preform, as illustrated in Figure 6. The employment of SAC305 solder instead of pure Sn led to an improvement in wettability and solderability. After bonding at 260 °C for 40 min under a pressure of 10.8 MPa, the bonded joint was composed of Cu-Sn IMCs and the residual Cu skeleton, indicating good thermal properties. The etched Cu foam brought enhanced joint strength due to its enlarged superficial area.

### 3.5. Cu-SnAgCu Molded Sheets

To address the issues of prolonged bonding time and void formation in TLP bonding, a dense Cu-SnAgCu molded sheet was fabricated through high-pressure (570 MPa) compaction at room temperature. This sheet was composed of spherical Cu and SAC particles [53]. In contrast to the non-pressure-molded mixed particles, the molded sheet exhibited an almost void-free structure. This characteristic facilitated the complete transformation of Sn into Cu_3_Sn. The shear strength of the bonded joint reached 50.5 MPa, which was 45% higher than that of the joint bonded with non-pressure-molded mixed particles. Moreover, the smooth surface of the compacted sheet effectively inhibited interfacial fracture propagation, demonstrating its potential as a void-free, high-temperature die attach material.

### 3.6. Cu-Sn Nanocomposite Interlayer (Cu-Sn NI)

**Figure 7:**
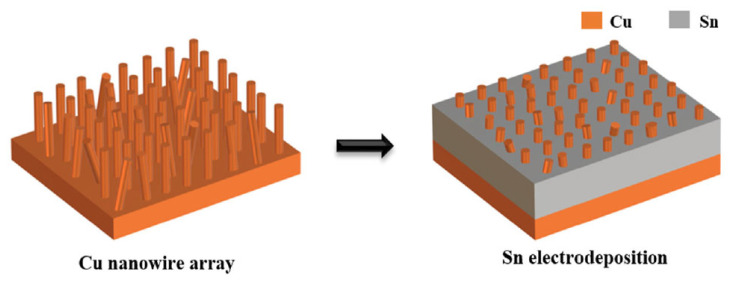
Schematic of the fabrication process of the Cu-Sn nanocomposite interlayer on the Cu substrate. Reprinted from Ref. [55].

Han Jiang et al. [32,55] designed and utilized a preformed Cu-Sn nanocomposite interlayer (Cu-Sn NI) for the bonding between Cu substrate and Cu bump. As shown in Figure 7, a regular array of Cu nanowires (200 nm diameter, 10 μm height) was deposited on Cu substrates. Subsequently, Sn was electrodeposited to fill the interwire gaps within the Cu nanowire array.

**Figure 8:**
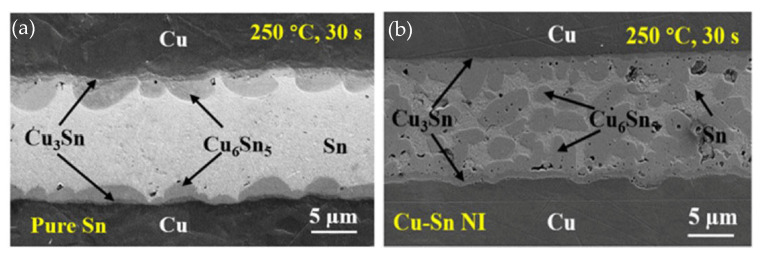
Microstructures of the joints after TLPB of (**a**) pure Sn and (**b**) Cu-Sn NI under a pressure of 5 MPa. Reprinted from Ref. [55].

In TLP bonding using a pure Sn interlayer, columnar Cu_6_Sn_5_ grains formed at the Cu/Sn interface, significantly impeding atomic diffusion between Cu and Sn [62]. Consequently, the reaction rate between Cu and Sn was slower, and it took about 60 min to achieve a full IMC joint. Microstructures of the TLPB joints bonded with pure Sn and Cu-Sn NI are shown in Figure 8. In contrast, all Cu nanowires were consumed during the initial stage of TLPB. Moreover, the Cu_6_Sn_5_ grains nucleated not only at the interfaces between the Cu substrates and interlayer, but also within the interlayer itself. This led to an acceleration of the IMC formation. As a result, full IMC joints were achieved in just 2 min. Due to the refinement of Cu-Sn IMC grains, the shear strength of the joints bonded with Cu-Sn NIs increased to 23.1 MPa, increasing ~29.1% compared with those bonded with pure Sn.

However, several defects were found within the brazed region. These included volume shrinkage voids, Kirkendall voids, detachment, and cracks induced by the CTE mismatch. To enhance the reliability of the joints further, effective solutions are urgently needed.

### 3.7. Sn-Cu Coated Multi-Walled Carbon Nanotubes (MWCNTs)

**Figure 9:**
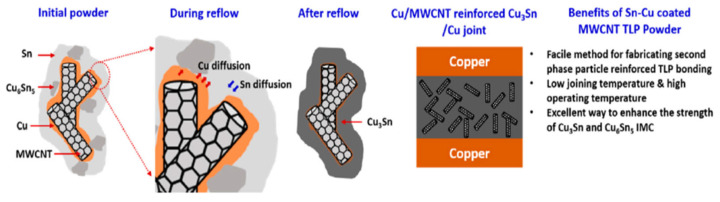
Schematic illustrating the mechanism of CuSn-MWCNT formation during TLP bonding. Reprinted from Ref. [33].

An innovative transient liquid-phase bonding material was prepared by consequent electroless plating of Cu and Sn on a multi-walled carbon nanotubes (MWCNTs) [33], as shown in Figure 9. During the plating process, a small amount of Sn reacted with Cu, leading to the formation of Cu_6_Sn_5_. The Sn-Cu coated MWCNT composite powders were blended with rosin to form a paste. Cu-Cu joints were bonded at 260 °C under a pressure of 10 MPa in air. A reaction time of 8 min was enough for the complete reaction between Cu and Sn. The joint consisted of MWCNT-reinforced Cu_3_Sn, and the shear strength reached 35.3 MPa, which was better than the traditional Cu/Sn foil/Cu joints.

### 3.8. Surface Structure Manipulation

For the traditional TLP bonding, electroplating is commonly employed for Cu metallization. The surface profile of Cu can be manipulated through formulating the plating solution.

**Figure 10:**
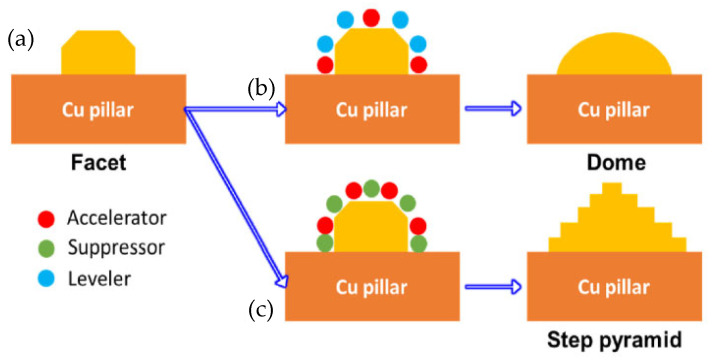
Growth mechanism of various structures on the surface of the Cu-electroplated layers: (**a**) facet, (**b**) dome and (**c**) step pyramid. Reprinted with permission from Ref. [56]. Copyright 2022 Elsevier.

**Figure 11:**
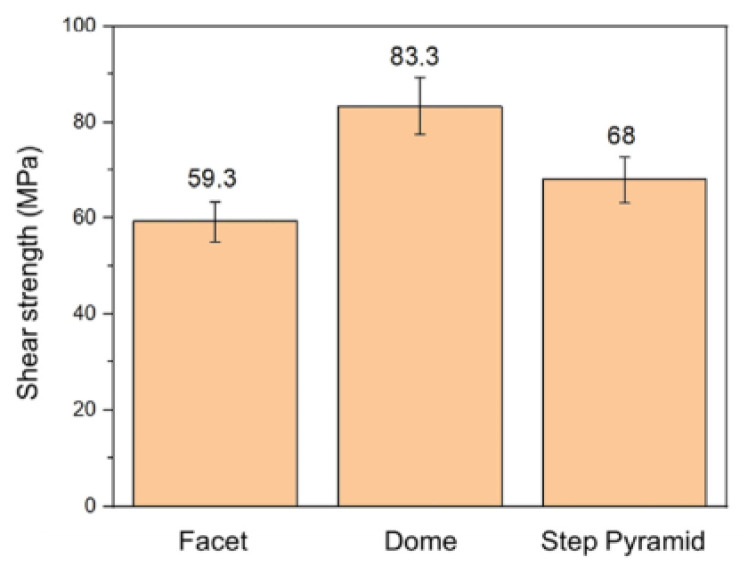
Shear strength of three TLP-bonded samples with the facet, dome, and step pyramid Cu surface structures. Reprinted with permission from Ref. [56]. Copyright 2022 Elsevier.

Hsu Shao-Yu et al. [56] investigated the influence of the surface profile of Cu on the Cu-Sn TLP bonding. As shown in Figure 10, surface structure of the Cu electroplated layer was manipulated by different additive formulas. These formulas were based on the addition of an accelerator, suppressor, and leveler to the plating solution. Three distinct crystal structures—a facet, dome, and step pyramid—were fabricated on the Cu surfaces and bonded with eutectic Sn-3.5 wt.% Ag solder alloy via TLP bonding at 260 °C for 20 min. The shear strength of the TLP-bonded joints is presented in Figure 11. The dome- and step pyramid-shaped surface structures effectively enhanced the bonding strength to 83.3 MPa and 68 MPa, respectively. This improvement in shear strength resulted from the riveting and interlocking effects of the unique surface geometries, which impeded crack propagation.

In the initial stage of TLPS, high-melting-point metal powder and low-melting-point metal powder were mixed to reduce the bonding time by utilizing the large surface area of the powders. However, problems such as the oxidation and non-uniformity of the mixed powder, high bonding pressure, and low joint strength impeded its practical application. Consequently, in the second stage, core–shell particles were proposed to overcome these problems. However, the required boding pressure was still high due to the thin Sn shell outside the Cu core.

Various innovative interlayers have since been proposed to address this issue. High-Sn-content Cu@Sn core/shell powder was prepared, and good bonding joints were achieved under a relatively low bonding pressure of 0.2 MPa. Nevertheless, the bonding time remained relatively long. An interlayer of Cu powders between Sn foils, a Sn-coated Cu foam preform, and Cu foam/SAC305 composite solder preform exhibited comparable connection qualities. Their preparations processes were simple. The Cu-Sn nanocomposite interlayer with Cu nanowire array embedded in Sn proved able to significantly decrease the bonding time to 2 min. However, there were voids, detachment, and cracks in the joint. Sn-Cu-coated MWCNT could achieve a full IMC joint in 8 min. Nevertheless, the high-temperature properties remained unknown, and the cost of this process was relatively high. The surface structure of the Cu substrate could be manipulated through the electroplating process, resulting in high joint strength. However, the bonding pressure was as high as 30 MPa, which may cause chip fracture. Although research into TLPS has demonstrated academic significance, substantial engineering challenges in bonding pressure, cost, and reliability need to be further investigated before practical industrial application.

## 4. Nanoparticle Sintering

Silver possesses the highest thermal conductivity (427 W/mK) among all metals and also exhibits resistant to oxidation [63]. In contrast to bulk and powder materials, nanoparticles exhibit a lower melting point due to the effect of their small size [64]. Favorable metallurgical joints can be achieved at relatively low temperatures while maintaining serviceability under high temperatures. The sintering temperature can be further reduced by decreasing the particle diameter and applying loading pressure. To prevent agglomeration, Ag nanoparticles are dispersed in organic solvents.

Ag nanoparticle sintered joints exhibit superior mechanical, thermal, and electrical properties, even under high service temperatures. Ag nanoparticles have been proven to be the most promising interconnect materials for WBG power semiconductor packaging [34]. However, the cost of silver remains relatively high. Furthermore, Ag exhibits electrochemical instability, which can lead to short circuits, particularly under conditions of high humidity or elevated temperatures. The high cost of silver, along with its electromigration issues, restricts its application in the packaging industry [65].

Copper exhibits electrical and thermal conductivity properties that are comparable to those of silver. However, its cost is substantially lower. As a result, Cu nanoparticles have attracted widespread attention in the field of electronic packaging [66,67]. Nevertheless, the strong oxidative tendency of Cu nanoparticles during sintering poses a substantial obstacle to their application. As a result, the sintering processes must be carried out under vacuum conditions [68], in an inert or reducing gas environment [25,69], or by utilizing reductive organic solvents [70].

In recent years, great efforts have been made to address the problems. Novel approaches were developed and are presented in Table 2.

### 4.1. Nanoparticle Paste

#### 4.1.1. Cu@Ag Nanoparticles

Silver suffers from the drawbacks of high cost and electric migration, whereas copper is susceptible to oxidation. To address these problems, a novel nanoparticle structure is proposed, which consists of a Cu particle as the core and a Ag shell on the outside, namely Cu@Ag nanoparticles. The Cu-Ag core–shell structure exhibits dual benefits. It not only reduces the migration and cost of nano-Ag but also alleviates the oxidation of nano-Cu.

**Figure 12:**
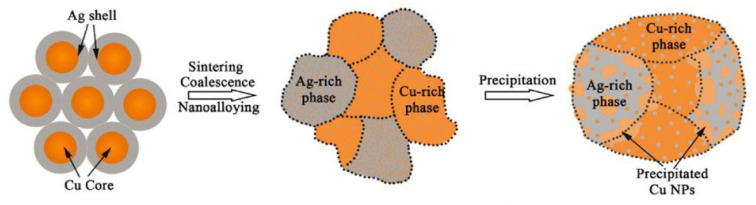
Schematic diagram of the low-temperature sintering mechanism of Cu@Ag NPs. Reprinted with permission from Ref. [35]. Copyright 2023 Elsevier.

Zhang Wenwu et al. [35] synthesized Cu@Ag NPs using chemical reduction via the polyol method and mixed them with 20 wt.% flux to prepare Cu@Ag NP pastes. Cu@Ag NP pastes were used to bond Cu and Cu. As the sample was sintered at 250 °C under a pressure of 5 MPa in a low-vacuum (0.5 Torr) atmosphere for 30 min, excellent shear strength (152 MPa) of the joint was achieved. A schematic diagram of the low-temperature sintering mechanism of Cu@Ag NPs is shown in Figure 12. The Cu core and Ag shell transformed into a supersaturated Ag-Cu nanoalloy containing interstitial solid solutions, Cu nanoprecipitates, ultrafine nanograin, low-angle grain boundaries, and twin boundaries. Consequently, through the combined strengthening effects of solid solution, secondary precipitated phases, and ultrafine nanograins, high-strength joints with excellent electro-thermal conductivity were successfully achieved for high-temperature electronic packaging applications.

However, the oxidation problem in the sintering process of Cu@Ag nanoparticles still existed due to the dewetting phenomena of Ag shell on the Cu core [81]. Wang Kaifeng et al. [71] synthesized Cu@Ag nanoparticles (~30 nm) via liquid-phase reduction. A new low-boiling-point mixed organic solvent was prepared by mixing 10% PEG-400, 10% terpineol, and 1% L-ascorbic acid. Then, Cu@Ag nanopastes were fabricated by mixing Cu@Ag nanoparticles with the organic solvent, which aimed to bond Cu-Cu joints under ambient air conditions. The joint exhibited good shear strength of 17.3 MPa when sintered at 250 °C for 30 min. There were still some voids in the joints, while full density was achieved at 300 °C for 30 min.

**Figure 13:**
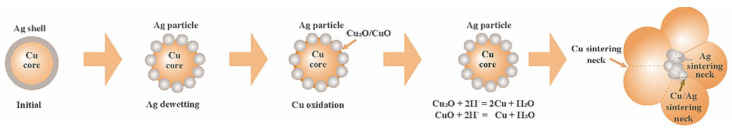
Micro sintering mechanism of Cu@Ag nanopaste. Reprinted with permission from Ref. [71]. Copyright 2024 Elsevier.

The sintering mechanism and antioxidant principle are demonstrated in Figure 13. As the heating temperature rose, the organic solvent on the surface of the Cu@Ag core–shell nanoparticle evaporated. Due to the high surface energy of the nanoparticle, dewetting occurred, accompanied by local sintering of the Ag shell. This led to the exposure and oxidation of the internal Cu core at high temperatures. L-ascorbic acid and PEG-400 in the solvent established a reducing condition, which reduced the oxidation of Cu. Finally, sintering necks such as Cu/Cu, Cu/Ag were established for the bonding.

#### 4.1.2. Self-Reducible Cu Nanoparticles

As discussed above, oxidation instability and the requirement for elevated sintering temperatures remain substantial challenges to conventional Cu nanoparticles. Yuan Yulei et al. [72] developed a novel self-reducible Cu nanoparticle paste to tackle these challenges. Under ambient conditions, Cu-Cu joints with excellent reliability and a high shear strength of 52.01 MPa were obtained at 250 °C under an applied pressure of 10 MPa without reducing agents or protective atmospheres. However, as bonding temperature further increased, the quality of the joints deteriorated due to the inevitable reoxidation of Cu nanoparticles.

**Figure 14:**
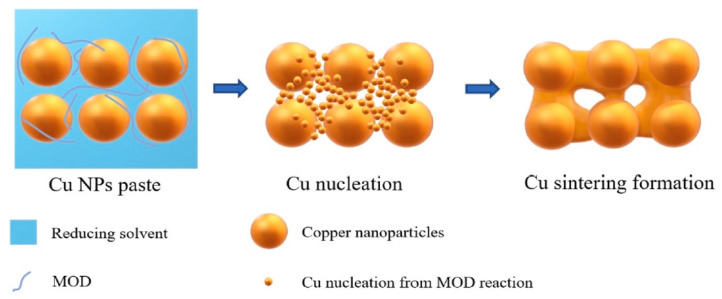
Schematic diagram of Cu-Cu sintering formation assisted by MOD. Reprinted with permission from Ref. [72]. Copyright 2021 Elsevier.

The self-reducible Cu nanoparticle paste was fabricated by mixing formic acid-treated Cu nanoparticles with reducing solvents and metal–organic decomposition (MOD) solutions. The MOD was complexed by copper formate and organic amines, which played an important role in the sintering process, as shown in Figure 14. The MOD solution performed dual functions of reducing surface oxides on Cu nanoparticles and concurrently nucleating new ultrafine Cu nanoparticles, thereby enhancing low-temperature sintering. Furthermore, the glucose additive in the reducing solvent effectively suppressed reoxidation of the Cu paste during both the storage and sintering stages, while ensuring sufficient Cu atomic diffusion.

#### 4.1.3. Liquid-Metal-Enhanced Nano/Microparticle Paste

Micro-sized and nano-sized particles were mixed to forming sintering paste, sufficiently playing their respective roles [82]. Nanoparticles can render the joint dense and improve joint performance. Microparticles can prevent particle agglomeration and crack formation during the sintering process. Li Jie et al. [83] prepared sintering paste by mixing Ag nanoparticles (20~100 nm) and microparticles (1~5 μm) according to the ratio of 1.5:1. Bare copper substrates were bonded at 265 °C in air without pressure. Nanoparticles have high surface energy, which facilitates the formation of sintering neck between nanoparticles and micron particles. Cu nano/microparticle pastes have attracted significant research attention for power electronic packaging applications. However, there are still inherent porosities in the joint, which damage joint performance.

**Figure 15:**
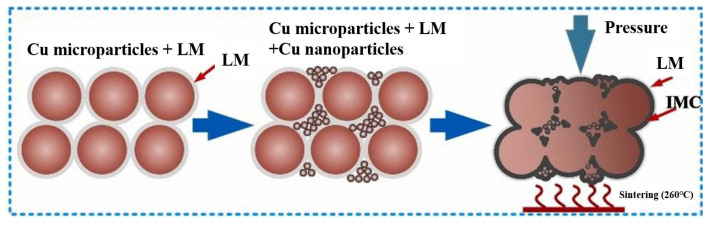
Sintering process of LM and Cu nano/microparticles. Reprinted with permission from Ref. [73]. Copyright 2023 Elsevier.

To address this issue, Liu Guangyin et al. [73] incorporated liquid metal (LM) into the Cu nano/microparticle paste solder. The LM exhibited outstanding wettability and flowability. It spontaneously infiltrated the voids within the nano/microparticle structure and established robust connections with the substrate surface, as shown in Figure 15. Eutectic Ga-In LM with a composition of 75.5 wt.% Ga and 24.5 wt.% In was used, and its melting point was 15.5 °C. LM was mixed with Cu microparticles and nanoparticles in different mass ratios. T2 Cu substrates were bonded at 260 °C for 30 min under a pressure of 4 MPa in atmospheric conditions. An appropriate mass ratio was important for the bonding quality. The shear strength reached its highest value of 27.5 MPa at a mass ratio of 60:40. The joints were mainly composed of CuGa_2_, In, Cu, and Cu_9_Ga_4_. Excessive LM content negatively affected thermal and electrical resistance due to the formation of LM droplets and excessive IMCs.

#### 4.1.4. Cu Nanoparticle Paste with Zn Powder

A good combination with high shear strength was fabricated by mixing Ag and Cu nanoparticles [84]. However, the effective mixing ratio of Ag had to exceed 50 wt.%, thus still resulting in a relatively high cost. Low-cost Zn has attracted attention due to its ability to form extensive solid solutions in copper (and at a remarkably lower temperature than the Cu liquid).

Toshikazu Satoh et al. [74] added Zn microparticle powders to Cu nanoparticle paste, with average sizes of 7 μm and 140 nm, respectively. The mixing ratio of Zn was varied between 0 and 70 wt.%. Multilayered Ag (100 nm)/Ni (200 nm)/Ti (100 nm) films were deposited on the SiC chips and DBC substrates to serve as adhesive layers. The joints were sintered in a H_2_ atmosphere. When the mixing ratio reached 30 wt.%, a 5 min sintering process at 250 °C achieved full Cu-Zn alloying across the bonding layer, resulting in peak bond strength (65.0 MPa). Furthermore, only a low pressure of 20 kPa was applied to fix the chips during sintering in this work, which avoided the risk of chip damage by the high pressure used in sintering conventional Ag nanoparticle paste.

#### 4.1.5. Bimodal-Sized Cu Nanoparticle Pastes

Traditional nano-copper paste is costly and is prone to oxidation, while submicron copper particles are low in price but have poor sintering activity. Therefore, a bimodal submicron-nano Cu paste composed of nano-Cu particles and submicron-Cu particles was proposed to improve sintering densification, increase thermal conductivity, and reduce cost [75]. The sintered joint was obtained under low pressure (0.4 MPa) in N_2_. The ratio of submicron-Cu and nano-Cu particles had a great influence on the bonding performance of Cu paste. The 1:1 mass ratio exhibited the best performance: 60.4 MPa shear strength, 11.3% porosity, and 11.2μΩ cm resistivity when sintered at 300 °C, which was significantly superior to the Cu-sintered joint of conventional nano-Cu pastes. Submicron particles play the role of raising packing density, suppressing cracks, and reducing cost. Nanoparticles act as a sintering bridge, enabling dense and low-shrink joints.

The bimodal-sized Cu nanoparticle paste was composed of 160 nm and 9 nm particles [76]. Small particles filled the porosities of big particles to promote densification. Using glycerol as the reducing solvent enables excellent atmospheric sintering of Cu paste, resulting in high-strength bonded joints. Glycerol suppresses oxidation, promotes densification, and forms ductile Cu bulks, demonstrating the paste’s suitability for high-power die attachment under mild conditions. Through Taguchi analysis, pressure was found to be the most important factor influencing the shear strength of the joint. Pressureless sintering at 280 °C/10 min achieved 22.3 MPa shear strength and 24% porosity, whereas sintering under 5 MPa raised shear strength up to 70.1 MPa and decreased the porosity to 4 %.

### 4.2. Organic-Free Nanoparticle Film

Nanoparticles (NPs) possess a high area-to-volume ratio and active surface energy. This characteristic leads to an impressively short bonding time (several minutes) and a high re-melting temperature (above 800 °C, approaching the melting point of the bulk metal) [85]. Simultaneously, organic stabilizing additives are incorporated to prevent the self-agglomeration and oxidation of nanoparticles [86,87]. To ensure the sufficient evaporation and decomposition of organic additives, the bonding process should be conducted at a high temperature above 250 °C. Moreover, the evaporation and decomposition of the organic solvent during the bonding process can generate voids in the joints, meaning they fail to meet reliability requirements for next-generation power electronics [88].

An organic-free bonding strategy is developed as a new die attachment solution, effectively preventing the aforementioned issues while avoiding the negative impacts of organic additives. Pure solid-state NPs are deposited as a surface modification layer on the final metallization layer of chips or substrates [89,90].

#### 4.2.1. Ag Nanoparticle Films

**Figure 16:**
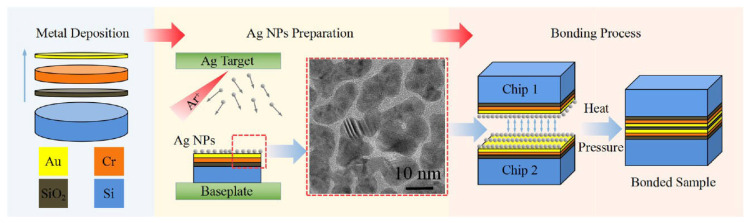
Fabrication process of Ag NPs for die attachment of power electronics (drawing not to scale). Reprinted with permission from Ref. [77]. Copyright 2022 Elsevier.

Fang JunPeng et al. [77] prepared Ag NPs on silicon wafers (with a 100 nm Cr/200 nm Au metallization layer) through high-pressure magnetron sputtering. The wafers were then diced into 10 mm × 10 mm chips. Au-Ag NP–Au bonding experiments were carried out at chip level, as shown in Figure 16. Reliable Au-Ag NP–Au bonding was achieved at a low temperature of 200 °C for 3 min under 20 MPa pressure. This phenomenon was attributed to sufficient interdiffusion of Au and Ag atoms, accompanied by recrystallization and grain growth at the bonding interface. The proposed bonding strategy enables rapid thermo-compression bonding at low temperatures, offering a new method for interconnecting technology.

**Figure 17:**
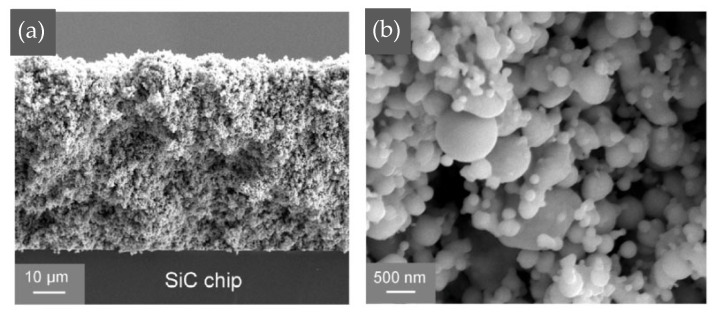
SEM image of deposited nanostructured film at (**a**) low magnification and (**b**) higher magnification. Reprinted with permission from Ref. [91]. Copyright 2020 Elsevier.

Wang Wengan et al. [91] fabricated an organic-free silver nanostructured film (SNF) on SiC chips (with 0.7 μm Ni/1 μm Ag metallization layer) via ultrafast pulsed laser deposition (PLD). The microstructure of the SNF film is shown in Figure 17. Large-sized particles were connected with necks, forming the “frame” structure. Meanwhile, small-sized particles formed branched clusters surrounding the “frame” particles, called “filler”. SiC chips with deposited SNF were directly bonded with the Ag-metallized (10 μm) substrates under assisted pressure at 180–300 °C. Since the SNF contained no organic solvent, the bonding temperature could be reduced to 180 °C while maintaining 18 MPa shear strength. As the bonding temperature was elevated to 250 °C, the average shear strength reached 40 MPa.

**Figure 18:**
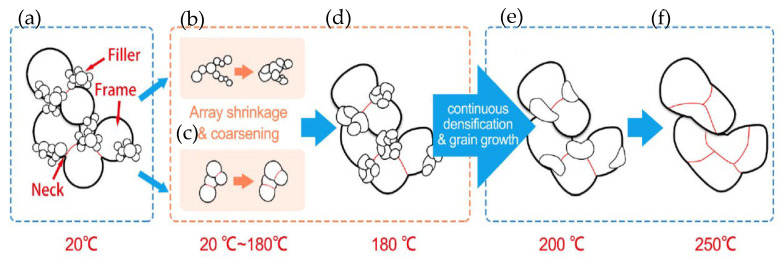
Schematic diagrams of densification and grain growth on “frame” and “filler” particles in SNF. (**a**) Initial stage at room temperature; (**b**,**c**) array shrinkage and coarsening process among the particles; (**d**–**f**) continuous densification and grain growth among the particles when the temperature increased from 180 °C to 250 °C. Reprinted with permission from Ref. [91]. Copyright 2020 Elsevier.

As depicted in Figure 18, the particles in SNF underwent densification and growth during the bonding process. Between temperatures of 20 °C and 180 °C, array shrinkage of “filler” particles and coarsening of “frame” particles occurred due to the high surface activity without organics. When the temperature increased from 180 °C to 250 °C, the continuous densification and grain growth among the particles took place.

#### 4.2.2. Ag-Pd Nanoalloy Film

Silver electrochemical migration limits the application of Ag nanoparticle sintering, especially in high-temperature power electronics [92,93]. As Ag-Pd alloy has been used as an electrode material to prevent electromigration, Pd was introduced to mitigate Ag migration during die attachment [94,95]. As the die attach material, Ag-Pb nanoparticles were deposited on the backside of the SiC power chip by pulsed laser deposition (PLD) at room temperature [36]. The Ag-Pd alloy target had a composition of 80 wt.% Ag and 20 wt.% Pd. The organics commonly used in traditional nanopastes were avoided.

**Figure 19:**
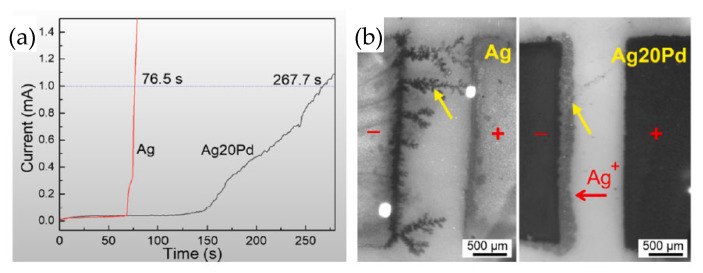
(**a**) Current vs. time during the water drop test; (**b**) electrochemical migration tests at 76.5 s. Reprinted with permission from Ref. [36]. Copyright 2021 Elsevier.

Low-temperature sintering at 300 °C for 5 min achieved excellent bonding quality, with a shear strength of 23.5 MPa. The sintered layer consisted of a Ag-Pd solid solution and a Ag-rich layer on the interface. The Ag-rich layer served as a prewetting medium for neck formation among Ag-Pd nanoparticles due to lower activation energy for Ag self-diffusion. The test of resistance to electrochemical migration is shown in Figure 19a. Taking 1 mA as the short-circuit current failure criterion, the short-circuit time for the Ag-20Pd nanoalloy was three times longer than that of the Ag nanoparticles. This indicates that the Ag migration was obviously inhibited. As shown in Figure 19b, dendritic Ag grew extensively on the pure Ag cathode, while growth was significantly suppressed on the Ag20Pd electrode under identical conditions.

#### 4.2.3. Cu Nanoparticle Film

Wu Yongchao et al. [37] prepared a Cu nanoparticle film (CNF) with a thickness of 10 μm for power electronic packaging using the same PLD method. There were a wide range of particle sizes. The existence of a large number of fine nanoparticles (with diameters smaller than 120 nm) greatly promoted sintering. Considering the high oxidative tendency of Cu nanoparticles, a reductive organic solvent, ethylene glycol (EG), was dropped onto the direct bonded copper (DBC) substrates before sintering in an inert atmosphere.

**Figure 20:**
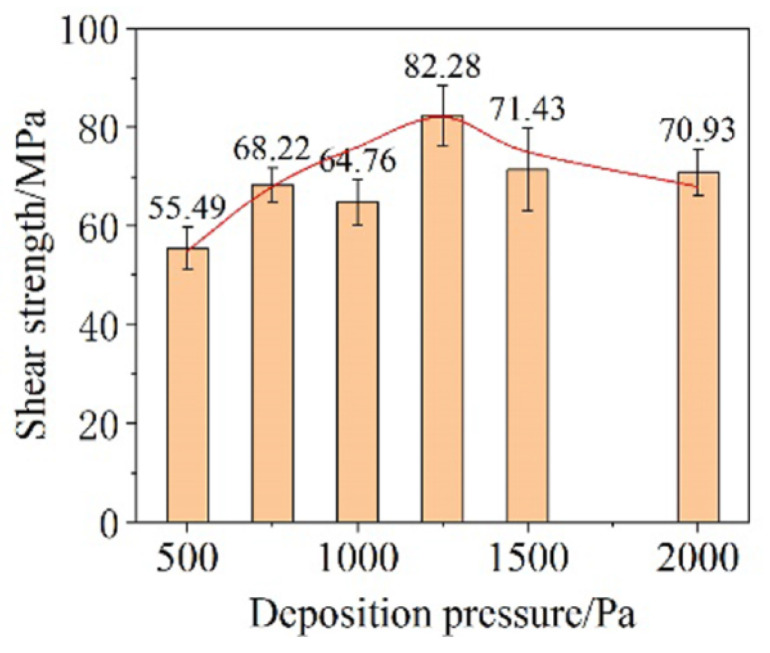
Shear strength of Cu-DBC joints using the CNF prepared under different deposition pressures. Reprinted with permission from Ref. [37]. Copyright 2022 Elsevier.

The influence of deposition pressure on bonding performance with parameters of 250 °C, 5 MPa, and 5 min was investigated. As the deposition pressure increased, the shear strength of the joints increased first and subsequently decreased (Figure 20). The turning point occurred at 1200 MPa. The proportion of small particles in CNF increased under the higher deposition pressure, promoting atoms’ diffusion during sintering. However, excessive deposition pressure resulted in high porosity in the film and poor necking among particles, which was detrimental to the bonding performance.

**Figure 21:**
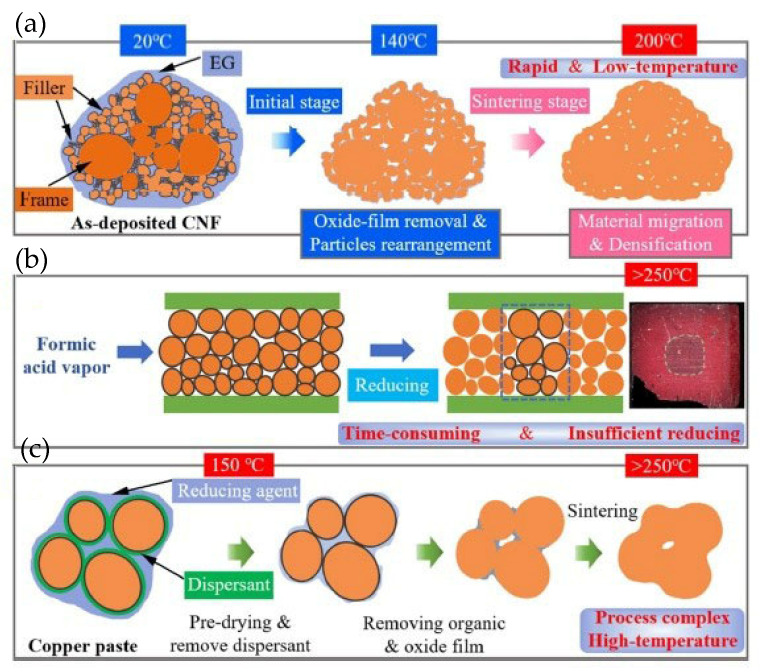
Comparison of three copper sintering methods. (**a**) Sintering process of CNF by EG reduction; (**b**) formic acid reduction sintering; and (**c**) sintering method of copper paste with reducing agent. Reprinted with permission from Ref. [37]. Copyright 2022 Elsevier.

As shown in Figure 21, sintering processes assisted by EG, formic acid, and reducing organics were comparatively analyzed. The CNF film exhibited a porous and loose structure. Consequently, the EG liquid infiltrated the CNF film via capillary action (Figure 21a). The oxide film of the nanoparticles was removed by EG at 140 °C, facilitating the formation of satisfactory joint strength at a temperature lower than 200 °C. The sintering process assisted by formic acid is illustrated in Figure 21b. Formic acid existed in the form of gas vapor. It required a relatively long time (more than 30 min) to reduce oxidization of the Cu particles. The sintering process of copper pastes with reducing organics is presented in Figure 21c. The reducing organics needed to decompose at temperatures above 200 °C. Therefore, the sintering temperature for the copper paste should be higher than 200 °C.

### 4.3. High-Thermal-Conductivity and Low-CTE Composite Paste

WBG power electronics generate a significant amount of heat during operation, and their performance and reliability are highly dependent on thermal conductivity and the coefficient of thermal expansion (CTE). Consequently, in the development of future interconnecting materials, high thermal conductivity for efficient heat dissipation and tunable CTE to minimize thermal stress will be taken into consideration.

#### 4.3.1. Ag/Diamond Composite Paste

Diamond has the highest thermal conductivity in nature (approximately 2200 W/(m·k) at 25 °C) and a relatively low coefficient of thermal expansion (CTE). Kähler [78] matched the CTE of the sintered Ag interconnect layer with the CTE of the thermoelectric material Bi_2_Te_3_ through adding diamond microparticles into Ag paste. Good bonding strength was obtained at 250 °C for 2 min under 6 MPa. The thermal conductivity of sintered Ag/diamond composites was lower than that of pure micro. The thermal conductivity of the intermediate connection layer is not only related to the inherent thermal conductivity of the composite paste itself but also to the porosity in the bonding layer.

**Figure 22:**
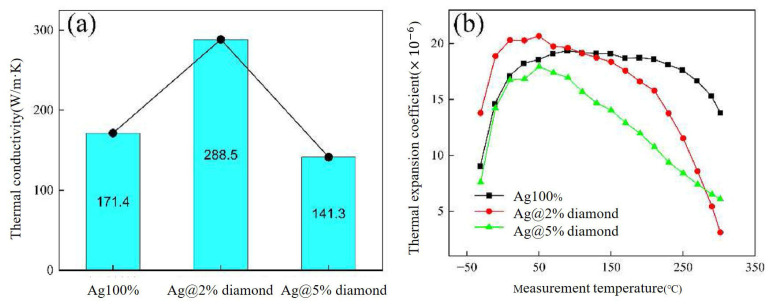
(**a**) The thermal conductivities and (**b**) CTE values of the Ag/diamond composite sintering pastes. Reprinted from Ref. [79].

Li Wangyun [79] mixed diamond nanoparticles with Ag flakes and CELTOL-IA to fabricate an Ag/diamond composite. The thermal conductivity of Ag@2%diamond paste increased to 288.5 W/m·K, while the CTE decreased to 11.5 × 10^−6^/°C, as shown in Figure 22. After 1000 cycles of thermal shock (−50–250 °C), the Ag@2% diamond joint retained 13.5% of its initial shear strength (vs. 10% for pure Ag) and exhibited suppressed Ag grain coarsening, reduced DBC substrate deformation, and fewer cracks. Excessive diamond (5 wt.%) led to particle agglomeration and higher porosity in the sintered joint. The Ag@2% diamond represents a successful diamond addition which balanced high thermal performance with thermomechanical stability.

#### 4.3.2. Ag/Si Composite Paste

**Figure 23:**
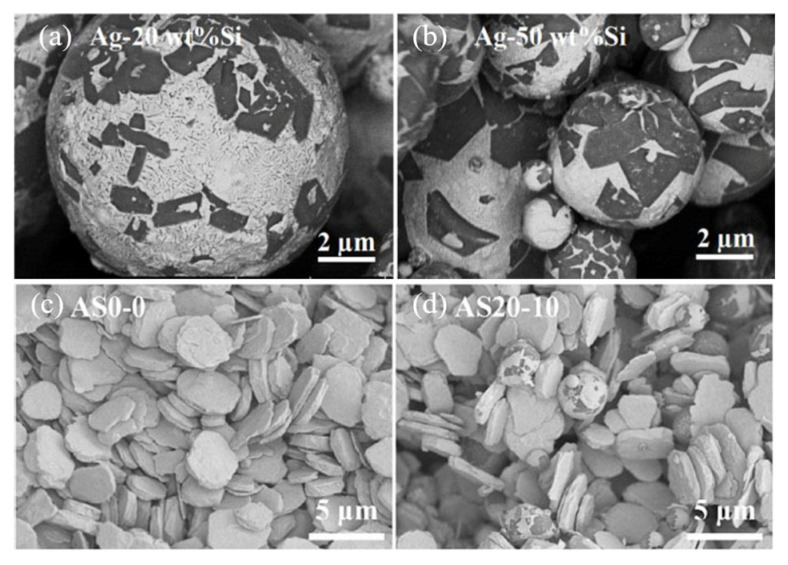
Morphology of Ag-Si atomized particles and paste: (**a**) Ag-20 wt.%Si; (**b**) Ag-50 wt.%Si; (**c**) Ag flakes; and (**d**) Ag20-Si10 paste. Reprinted from Ref. [80].

The difference in CTE among the Ag (Ag 19.7 × 10^−6^/°C), SiC (4.4 × 10^−6^/°C), GaN (5.6 × 10^−6^/°C), and DBC substrate (16.5 × 10^−6^/°C) induced thermal stress in the joint, which resulted in chip cracking. Si particles were added into Ag paste to reduce CTE [80]. Firstly, Ag-20 wt.%Si and Ag-50 wt.%Si particles were fabricated by atomization, as shown in Figure 23a,b. Then Ag-Si atomized particles and micro Ag flakes (Figure 23c) were mixed as a composite paste according to different Si contents of 10 vol% and 30 vol% (Figure 23d). To produce Ag20-Si10, Ag-20Si was mixed with Ag flakes, and the Si content was 10%. As the Ag-Si atomized particle content increased, CTE decreased (from 19.7 × 10^−6^/°C of pure Ag to 12.8 × 10^−6^/°C of AS20-30).

**Figure 24:**
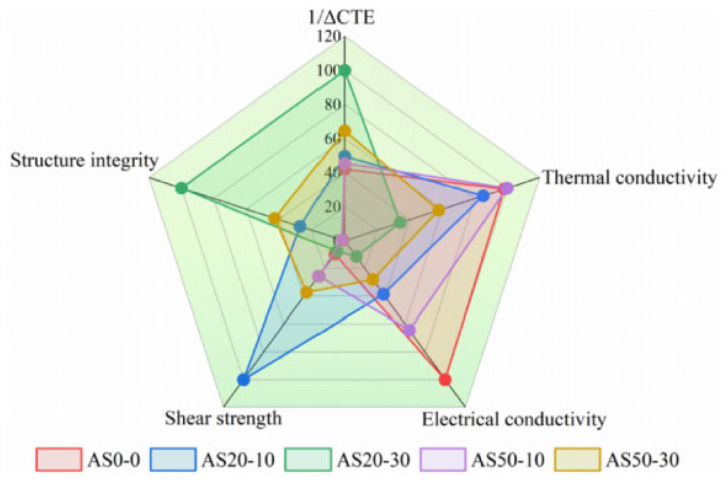
Radar chart of the critical material properties of the pastes and factors affecting the reliability of sintered joints. Reprinted from Ref. [80].

Sintering joints between SiC chips and DBC substrates were achieved at 300 °C for 60 min under an assisted pressure of 20 MPa. After a thermal shock test (−50~250 °C) for 100, 500, and 1000 cycles, the thermal conductivity, CTE, tensile strength, and shear strength of each paste were investigated. As shown in Figure 24, radar chart assessment identified AS20-10 and AS50-30 as the best-balanced candidates, providing reliable die attach solutions with low CTE for long-life SiC power modules.

Cu@Ag core–shell nanoparticles have emerged as a promising strategy with which to impede the oxidation of Cu and mitigate the migration of Ag. Nevertheless, the efficacy of this approach is weakened by the dewetting phenomenon of the Ag shell on the Cu core. The presence of a reducing solvent remained indispensable during the sintering process. Moreover, the self-reducible Cu NP paste allowed the sintering process to occur in air. The MOD transformed into ultrafine Cu nanoparticles, which was beneficial for the low-temperature sintering of Cu. The liquid metal and Zn microparticle powder were employed as filler metals to infiltrate the porosities among Cu particles, thereby reducing voids in the joint and associated costs.

Another promising advancement in nanoparticle sintering is the organic-free bonding strategy. An organic-free nanoparticle film was deposited on the substrate. There was no organic solvent to volatilize and decompose. Therefore, the bonding temperature was decreased to 140–200 °C, and no voids were generated from organic solvents. Pd was added into the Ag nanoparticle film, which effectively inhibited the electromigration of Ag. The morphology and thickness of the film are determined by the deposition parameters, and these characteristics in turn exert an influence on the bonding strength.

The incorporation of diamond and Si additives offers a versatile approach for adjusting the thermal conductivity and CTE of Ag paste. Nevertheless, the bonding process necessitates modification, owing to the presence of voids in the sintered joint.

## 5. Conclusions

Within the development and application of third-generation wide-bandgap (WBG) power electronics, we note inevitable trends. There is increasing demand for novel high-temperature packaging technologies with excellent properties. Traditional approaches to addressing the problems of long bonding time, voids, and powder oxidation have largely involved using a thinner interlayer for TLP, Cu@Sn core/shell powder forTLPS, and Ag@Cu core/shell particles for nano-Ag sintering. Herein, we have comprehensively reviewed innovative materials and methods for TLP, TLPS, and nano-Ag sintering presented over the past five years.

Ni and SiC NWs have been added into the interlayer to control the formation of IMCs and voids in the joint. The structure of the interlayer is no longer confined to laminated multilayers or mixed powders. Instead, porous foams, nanowires, and multi-walled carbon nanotubes (MWCNTs) have been employed to enhance the contact area between low-melting-point and high-melting-point materials, thereby improving the shear strength.

Organic-free nanoparticle films provided a novel way of avoiding the negative impacts generated from organic additives. Ag composite pastes with Si and diamond additives are effective in adjusting thermal conductivity and the CTE, meaning they meet the requirements of WBG power electronics.

From the perspective of engineering applications, ideal interconnecting materials for power device packaging are required to satisfy the following crucial performance criteria.

(1) Production efficiency and yield

Appropriate bonding time is an important factor for interconnection materials employed in commercial applications. Many studies have been devoted to reducing the bonding time from 150 min to 2 min. When the bonding time exceeds 20 min, production efficiency appears to be inadequate for industry application.

To ensure bonding strength, it is generally necessary to apply pressure to the chip during TLP, TLPS, and nanoparticle sintering processes. However, high pressure (exceeding 5 MPa) may cause damage to or fracture wide-bandgap (WBG) chip. Therefore, the bonding pressure of some interconnection materials needs to be further reduced.

The interface between high-power wide-bandgap (WBG) chips and the substrate demands superior low-ohmic contact and efficient thermal management. The porosity of the connection layer should be maintained below 10% according to GJB548B. This necessitates that the interconnection materials exhibit excellent wettability on the substrate.

(2) Strength and high-temperature reliability

For WBG electronics, the operating temperature can reach over 300 °C under working conditions. As of today, various studies on shear strength during high-temperature aging have been carried out. However, there remains a dearth of reliability verification under the synergistic influence of thermal and electrical factors.

(3) Thermal conductivity and coefficient of thermal expansion (CTE)

High-power WBG electronics generate a large amount of heat during operation, which must be dissipated efficiently. When serving as the thermal interface material, the thermal conductivity of the interconnecting materials represents a crucial performance parameter. Moreover, the coefficients of thermal expansion (CTEs) of substrates, chips, and interconnect materials are different. This discrepancy can lead to stress concentration and even chip cracking during high-temperature operation.

Currently, research primarily focuses on bonding processes and mechanical strength. Despite silver and copper exhibiting excellent thermal conductivity, only a limited number of studies focus on the thermal conductivity and CTE matching of the interconnect materials. The addition of Si and diamond into Ag paste can adjust thermal conductivity and the CTE. However, porosity and cracking in the sintered region should be alleviated.

(4) Thermal shocking and thermal cycling

There are temperature fluctuations and current switching during the service life of WBG electronics. Consequently, CTE mismatch among substrate, chip, and interlayer materials induces thermomechanical stress. More studies have to be carried out after thermal shocking and thermal cycling tests.

(5) Gas emission

After transient liquid-phase sintering and nanoparticle sintering, residual organics still remain in the joint. These organics can release gas under high temperatures during the operation of WBG devices. Particularly for sealed components, gas emission may cause the breakdown of electronics inside the components.

(6) Economic efficiency

Once performance meets the aforementioned requirements, the cost of new interlayer materials should be competitive in accordance with the market. Typically, the cost should not exceed 1.5 times that of current materials.

Novel interconnecting materials are proposed as promising die attach materials for WBG power electronics. Nevertheless, existing solutions still encounter several challenges. These include low production efficiency and yields that need to be enhanced, high costs that require reduction, and a shortage of studies validating long-term and high-temperature reliability. In the future, packaging design must co-optimize electrical, thermal, and mechanical performance, leveraging AI and simulation tools to enable intelligent design.

## Figures and Tables

**Table 1:** New approaches recently proposed for TLPS.

New Approaches	Sintering Parameters (°C-min-Mpa)	Atmosphere	Shear Strength /MPa	Advantages	References
High-tin-content Cu@Sn core/shell powder	300-150-0.2	vacuum	20.67	sufficient liquid Sn to fill porosities	[22]
Interlayer of Cu powders between Sn foils	300-45-0.3.	air	32.9	sufficient liquid supplements for the original gaps	[54]
Sn-coated Cu foam preform	280-40-2	Ar	28.1	complete infiltration of the low-melting-phase material into the foam’s porous matrix	[30]
Cu foam/SAC305 composite solder preform	260-40-10.8	air	53.9	improve the wettability of Sn on Cu foam	[31]
Cu-SnAgCu molded sheets	300-20-5	formic acid	50.5	highly dense without voids; smooth surface	[53]
Cu-Sn nanocomposite interlayer	250-2-5	air	23.1	Cu_6_Sn_5_ grains nucleate inside the interlayer leading a short reaction time for full IMC joints	[32,55]
Sn-Cu coated MWCNT	260-8-10	air	35.3	interfacial contact area of Cu/Sn increases and the joint is reinforced by MWCNT	[33]
Surface structure manipulation	260-20-30	air	68/83.3	riveting and interlocking effects suppress crack propagation	[56]

**Table 2:** Comparison of various nanoparticle (NP) sintering processes and performance.

Interlayer Materials		Sintering Parameters (°C-min-Mpa)	Atmosphere	Shear Strength/MPa	Advantage	References
Nanoparticle paste	Cu@Ag NPs paste	250-30-5	vacuum	152	Oxidation resistance	[35]
		250-30-10	air	17.3	Oxidation resistance	[71]
	Self-reducible Cu NP paste	250-20-10	air	52.01	Oxidation resistance	[72]
	Liquid metal-enhanced NP paste	260-30-4	air	27.5	Void-free	[73]
	Cu NP paste with Zn powder	350-5-0.02	H_2_	65	Pressureless	[74]
	bimodal submicron–nano Cu paste	300-30-0.4	N_2_	60.4	High-density Low-cost	[75]
	bimodal Cu-nanoparticle paste	280-10-0	air	22.3	No pressure or pressureless	[76]
		280-10-5		70.1		
Organic-free nanoparticle film	Ag NP film	200-3-20	air	10	Organic-free	[77]
	Ag-Pb nanoalloy film	300-30-20	air	23.5	Organic-free Ag migration inhibit	[36]
	Cu NP films	140-5-5	EG+ Ar	22	Organic-free	[37]
		250-5-1		50–65	Low-cost	
High-thermal-conductivity and low-CTE composite paste	Ag/Diamond composite paste	250-2-6	air	10.2/6.2	Low-CTE	[78]
		250-60-0	air	43.8/67.5	Low-CTE High thermal conductivity No pressure	[79]
	Ag/Si composite paste	300-60-20	air	80–100	Low-CTE	[80]

## Data Availability

No new data were created or analyzed in this study. Data sharing is not applicable to this article.
